# Supplementary material for: Finely tuned eye movements enhance visual acuity
Source: Nat Commun. 2020 Feb 7;11:795. doi: 10.1038/s41467-020-14616-2 (PMC7005897; doi:10.1038/s41467-020-14616-2)
Supplement: Supplementary file 1 — Supplementary Information [file 41467_2020_14616_MOESM1_ESM.pdf]

Supplementary Information

Finely tuned eye movements enhance visual acuity

Janis Intoy and Michele Rucci

Updated: January 6, 2020

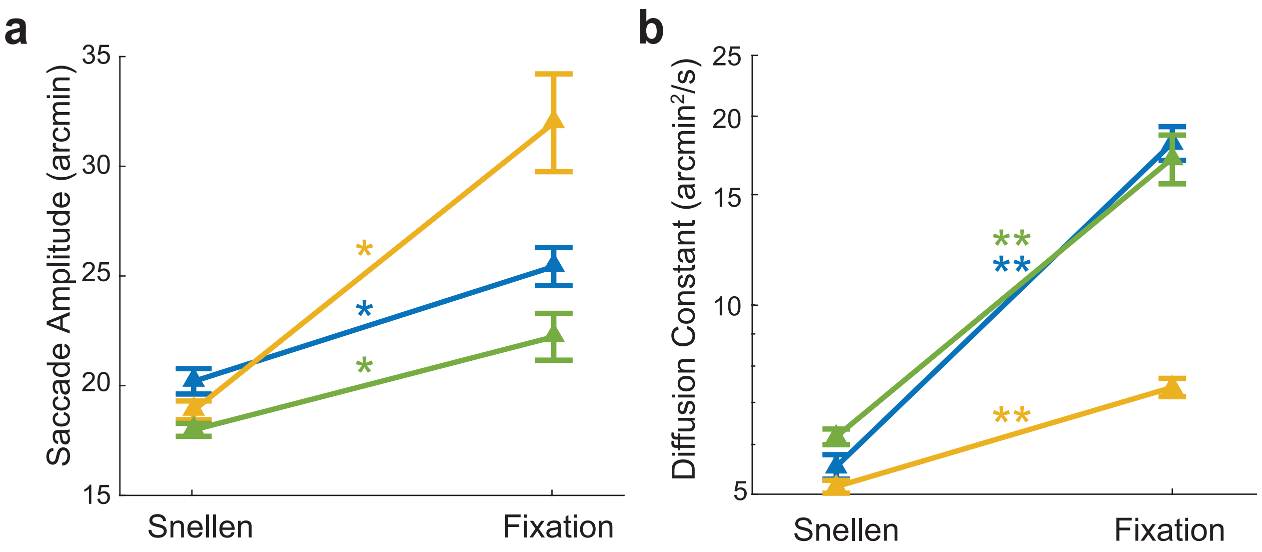

Supplementary Figure 1. Oculomotor characteristics during isolated fixation. The eye movements data collected in the Snellen test are compared to those acquired in a separate experiment, in which subjects were asked to maintain accurate fixation at the center of the display for the entire duration of the trial (Fixation condition). The fixation dot was presented at full contrast over a uniform gray background. (**a**) 90th percentiles of the saccade amplitude distributions and (**b**) average drift diffusion constants measured in the two conditions. Data are shown for three subjects, represented here by different colors. Error bars represent bootstrap SEM ($^{*}p < 0.001$, two-tailed two-sample Kolmogorov-Smirnov test; $^{**}p < 0.001$, two-tailed two-sample permutation test). Source data are provided as a source data file.

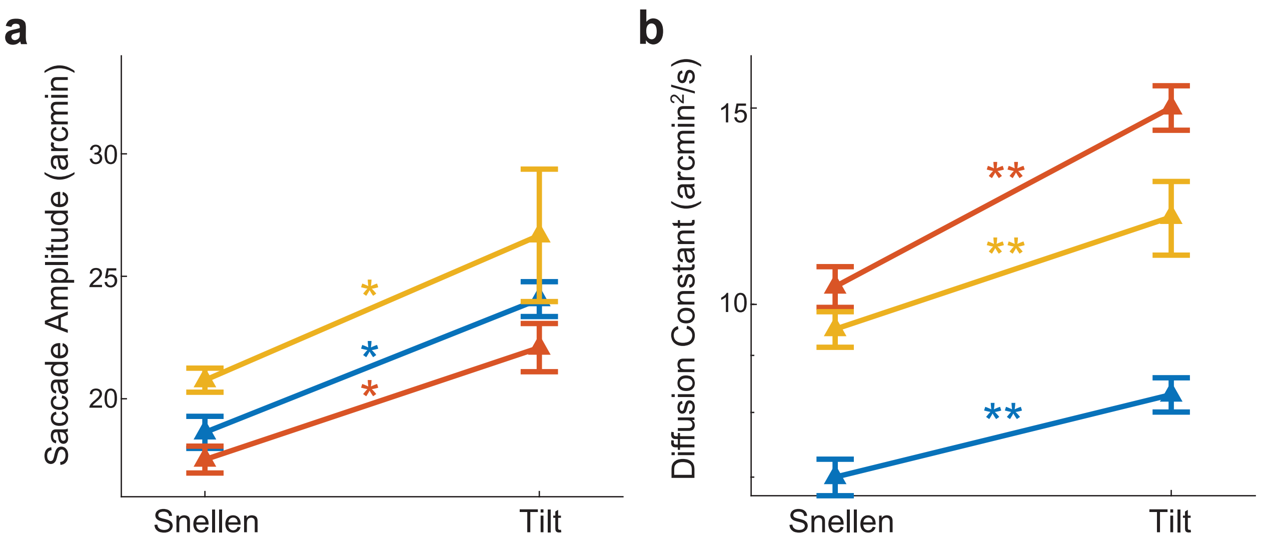

Supplementary Figure 2. Task dependence of oculomotor activity. The oculomotor data collected in the Snellen test are compared to those acquired when observers performed a non-acuity task with the same stimuli. Subjects now reported whether the entire 0 logMAR line of the Snellen eye chart was tilted by ±4° ("Tilt" condition). (**a**) 90th percentiles of saccade amplitude distributions and (**b**) average drift diffusion constants measured in the two conditions. Data are shown for three subjects, represented by different colors. Error bars represent bootstrap SEM ($^{*}p < 0.001$, two-tailed two-sample Kolmogorov-Smirnov test; $^{**}p \leq 0.003$, two-tailed two-sample permutation test). Source data are provided as a source data file.

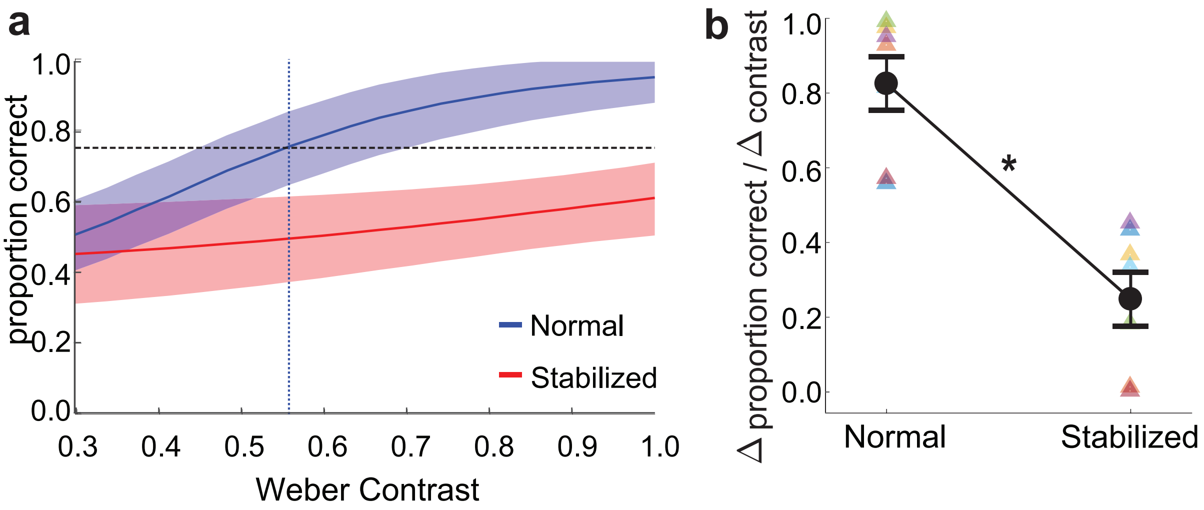

Supplementary Figure 3. Average performance as a function of stimulus contrast. **(a)** In the presence of fixational drift, correct identification strongly depends on contrast (blue curve). This is not the case under retinal stabilization (red curve). With stabilized stimuli, threshold performance (dashed line) could not be recovered even at maximum contrast. Stimuli were 0 logMAR optotypes. Shaded regions represent SEM. **(b)** Sensitivity to contrast changes at threshold. Circles represent the average slopes of the psychometric functions at the contrast threshold level (dotted line in *a*). Error bars represent SEM. Triangles are the individual subject data ($^*p = 0.016$, two-tailed Wilcoxon signed-rank test). Source data are provided as a source data file.

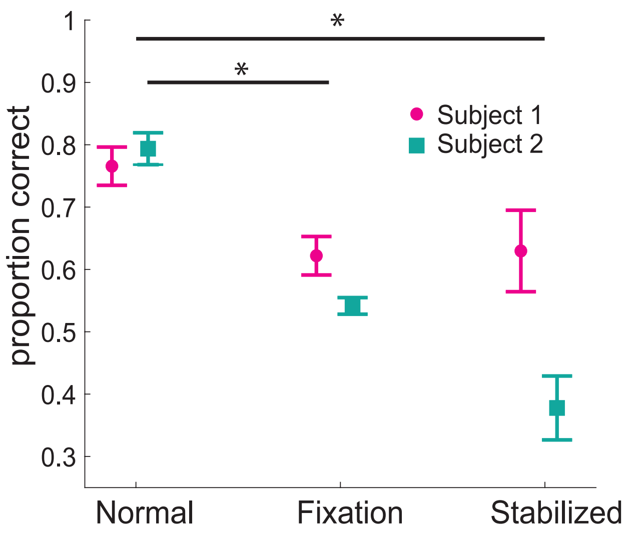

Supplementary Figure 4. Consequence of suppressing of the microsaccade sequence. Performance in the Snellen task is impaired when subjects are instructed to maintain fixation at the center of the array, a condition in which the normal microsaccade sequence is suppressed (Fixation condition). For comparison, performance measured under retinal stabilization are also shown (Stabilized). Data points represent average proportions of correct responses for two subjects. Error bars are SEM ($^{*}p < 0.05$, one-tailed Z-test for each individual). Source data are provided as a source data file.
