# Supplementary material for: Finely tuned eye movements enhance visual acuity
Source: Nat Commun. 2020 Feb 7;11:795. doi: 10.1038/s41467-020-14616-2 (PMC7005897; doi:10.1038/s41467-020-14616-2)
Supplement: Supplementary file 3 — Reporting Summary [file 41467_2020_14616_MOESM3_ESM.pdf]

nature research

Corresponding author(s): Michele Rucci
Last updated by author(s): Dec 31, 2019

Reporting Summary

Nature Research wishes to improve the reproducibility of the work that we publish. This form provides structure for consistency and transparency in reporting. For further information on Nature Research policies, see Authors & Referees and the Editorial Policy Checklist .

Statistics

For all statistical analyses, confirm that the following items are present in the figure legend, table legend, main text, or Methods section.

n/a	Confirmed	
☐	☒	The exact sample size (n) for each experimental group/condition, given as a discrete number and unit of measurement
☐	☒	A statement on whether measurements were taken from distinct samples or whether the same sample was measured repeatedly
☐	☒	The statistical test(s) used AND whether they are one- or two-sided<br>*Only common tests should be described solely by name; describe more complex techniques in the Methods section.*
☐	☒	A description of all covariates tested
☐	☒	A description of any assumptions or corrections, such as tests of normality and adjustment for multiple comparisons
☐	☒	A full description of the statistical parameters including central tendency (e.g. means) or other basic estimates (e.g. regression coefficient) AND variation (e.g. standard deviation) or associated estimates of uncertainty (e.g. confidence intervals)
☐	☒	For null hypothesis testing, the test statistic (e.g. F, t, r) with confidence intervals, effect sizes, degrees of freedom and P value noted<br>*Give P values as exact values whenever suitable.*
☒	☐	For Bayesian analysis, information on the choice of priors and Markov chain Monte Carlo settings
☒	☐	For hierarchical and complex designs, identification of the appropriate level for tests and full reporting of outcomes
☒	☐	Estimates of effect sizes (e.g. Cohen's d, Pearson's r), indicating how they were calculated

Our web collection on statistics for biologists contains articles on many of the points above.

Software and code

Policy information about availability of computer code

Data collection	Eye movement traces and subject responses were collected by means of EyeRIS, a custom hardware and software system for acquiring data and controlling stimulus display and timing (Santini et al, 2007). This system has been extensively tested and previously described (Rucci et al., 2007; Poletti et al., 2013).
Data analysis	Data were analyzed in Matlab. Segmentation and characterization of recorded eye traces using algorithms described in the literature (Cherici et al., 2012; Poletti et al, 2013; Yu et al., 2018).

For manuscripts utilizing custom algorithms or software that are central to the research but not yet described in published literature, software must be made available to editors/reviewers. We strongly encourage code deposition in a community repository (e.g. GitHub). See the Nature Research guidelines for submitting code & software for further information.

Data

Policy information about availability of data

All manuscripts must include a data availability statement. This statement should provide the following information, where applicable:
- Accession codes, unique identifiers, or web links for publicly available datasets
- A list of figures that have associated raw data
- A description of any restrictions on data availability

The source data underlying Figs 2, 3a-c,e, and 4b-d and Supplementary Figs 1-4 are provided as a Source Data file. All other data supporting the findings reported here are available upon reasonable request from the corresponding author.

Field-specific reporting

Please select the one below that is the best fit for your research. If you are not sure, read the appropriate sections before making your selection.

☒ Life sciences ☐ Behavioural & social sciences ☐ Ecological, evolutionary & environmental sciences

For a reference copy of the document with all sections, see nature.com/documents/nr-reporting-summary-flat.pdf

Life sciences study design

All studies must disclose on these points even when the disclosure is negative.

Sample size	The sample size of naive subjects (n=7) was chosen to ensure that significant effects generalize to a majority of the population when exhibited by each individual (see Anderson & Vingrys, 2001). Note, however, that we are primarily interested in effects within individuals, not about population means. Each individual datum is based on hundreds of trials. Additional subjects completed control experiments (n=92) described in the Methods.
Data exclusions	No subjects were excluded from this study. The exclusion of data from individual trials are described in Methods:Oculomotor data.
Replication	Experimental findings were replicated across all individual observers in the study.
Randomization	Each subject participated in all experimental conditions. Trials were blocked by condition and completed in random order.
Blinding	Investigators were not blinded to experimental conditions so as to provide appropriate instructions to subjects.

Reporting for specific materials, systems and methods

We require information from authors about some types of materials, experimental systems and methods used in many studies. Here, indicate whether each material, system or method listed is relevant to your study. If you are not sure if a list item applies to your research, read the appropriate section before selecting a response.

Materials & experimental systems

n/a	Involved in the study	
☒	☐	Antibodies
☒	☐	Eukaryotic cell lines
☒	☐	Palaeontology
☒	☐	Animals and other organisms
☐	☒	Human research participants
☒	☐	Clinical data

Methods

n/a	Involved in the study	
☒	☐	ChIP-seq
☒	☐	Flow cytometry
☒	☐	MRI-based neuroimaging

Human research participants

Policy information about studies involving human research participants

Population characteristics	See Methods paragraph #1.
Recruitment	Subjects were recruited by means of flyers and advertisements posted on the Boston University campus and online. The majority of subjects are undergraduate students and reflect the population distribution of students at the University. Additional subjects who participated in control experiments were similarly recruited at the University of Rochester.
Ethics oversight	The study protocol was approved by the Boston University Charles River Campus Institutional Review Board and the Research Subjects Review Board at the University of Rochester.

Note that full information on the approval of the study protocol must also be provided in the manuscript.
