# Supplementary material for: Finely tuned eye movements enhance visual acuity
Source: Nat Commun. 2020 Feb 7;11:795. doi: 10.1038/s41467-020-14616-2 (PMC7005897; doi:10.1038/s41467-020-14616-2)
Supplement: Supplementary file 4 — Description of Additional Supplementary Files [file 41467_2020_14616_MOESM4_ESM.pdf]

Description of Additional Supplementary Files

File Name: Supplementary Movie 1
Description: **Example of eye movements.** An example of high-resolution oculomotor recording during examination of the 20/20 line. (Top panel) The center of gaze (cross) is shown superimposed on the observed row of tumbling-E optotype. Pink and green segments refer to periods of microsaccades and drifts, respectively. (Bottom panel) The same traces shown as a function of time.

File Name: Supplementary Movie 2
Description: **Simulated neural responses.** Pattern of activity in an array of modeled P ganglion cells exposed to the reconstructed luminance flow on the retina during eye drift. The two panels show simulations for a larger (left) and smaller (right) drift. Levels of simulated activity are color-coded so that red and blue represent high and low responses, respectively. Note that the smaller drift measured in the Snellen test enhances the relevant edges of the optotype.

File Name: Supplementary Movie 3
Description: **Dynamics of gaze.** Average probability distribution of gaze position during the course of a trial. The 5 arcmin squares mark the positions of the six optotypes. Darker colors represent higher probabilities to find gaze in a given location. Note that the distribution shifts progressively rightwards as the trial progresses.
